# Fatal Arrhythmic Complications of Multisystemic Inflammatory Syndrome (MIS-C) in a Pediatric Patient

**DOI:** 10.7759/cureus.60927

**Published:** 2024-05-23

**Authors:** Chika C Oragui, Arthur Dilibe

**Affiliations:** 1 Pediatrics, Pediatric Intensive Care Unit (PICU), Stanford University School of Medicine, Lucile Packard Children's Hospital, Palo Alto, USA; 2 Internal Medicine, ECU Health, East Carolina University, Greenville, USA

**Keywords:** multisystemic inflammatory syndrome associated with covid -19 (mis-c), coronavirus disease of 2019 (covid-19), bigeminy, av blocks, arrhythmias

## Abstract

In 2019, the emergence of the coronavirus disease 2019 (COVID-19) virus triggered a global pandemic, reminiscent of the magnitude witnessed during the flu pandemic of 1918. Initially, children often presented with either asymptomatic or mild upper respiratory tract infection symptoms. However, in the post-acute phase, a distinct syndrome affecting multiple organ systems emerged, sharing similarities with Kawasaki's disease. This syndrome was later classified as multisystem inflammatory syndrome in children (MIS-C) by the Pediatric Intensive Care Society in April 2020. Notably, cardiac manifestations and complications associated with COVID-19 constitute a significant source of morbidity and mortality, characterized by left ventricular dysfunction, cardiac conduction abnormalities, and arrhythmias. Although cases of arrhythmias with MIS-C are rare in the literature, we present a unique case involving a 14-year-old without known cardiac risk factors who presented with conduction abnormalities and fatal arrhythmias secondary to MIS-C.

## Introduction

In 2019, the emergence of the coronavirus disease 2019 (COVID-19) virus triggered a global pandemic, reminiscent of the magnitude witnessed during the flu pandemic of 1918. Initially, children often presented with either asymptomatic or mild upper respiratory tract infection symptoms. However, in the post-acute phase, a distinct syndrome affecting multiple organ systems emerged, sharing similarities with Kawasaki's disease. This syndrome was later classified as multisystem inflammatory syndrome in children (MIS-C) by the Pediatric Intensive Care Society in April 2020 and can be described as a post-infectious hyperinflammatory disorder associated with COVID-19 causing a wide spectrum of systemic inflammation typically presenting with fever, shock, and an array of cardiac dysfunctions [1-5]. Notably, the cardiac manifestations and complications associated with COVID-19 constitute a significant source of morbidity and mortality, characterized by left ventricular dysfunction, cardiac conduction abnormalities, and arrhythmias. Cardiac involvement occurs in up to 60-80% of children with MIS-C and is more common in MIS-C than in Kawasaki disease (KD) [5,6]. While cases of arrhythmias with MIS-C are rare in the literature, we present a unique case involving a 14-year-old without known cardiac risk factors who presented with conduction abnormalities, subsequently developed fatal arrhythmias, and unfortunately went into cardiac arrest as a complication of MIS-C.

## Case presentation

A 14-year-old female with a medical history of intermittent asthma (albuterol 90 mcg/actuation inhaler as needed) presented to the emergency department with a five-day duration of fever, nausea, vomiting, epigastric pain, retrosternal chest discomfort, and shortness of breath. Her recorded temperature peaked at 103°F (39.4°C). The patient localized her chest discomfort to the sternal and epigastric regions, reporting an association with dyspnea at rest and nausea. Emesis was described as non-bilious and non-bloody. Initial evaluation by her primary care physician revealed unremarkable findings, including a negative COVID-19 test, prompting supportive management. However, fever persistence, worsening symptoms, and poor oral intake led to her presentation to the emergency department. The patient denied recent domestic or international travel but reported recent contact with her brother, diagnosed with COVID-19 infection one month prior.

**Figure 1:**
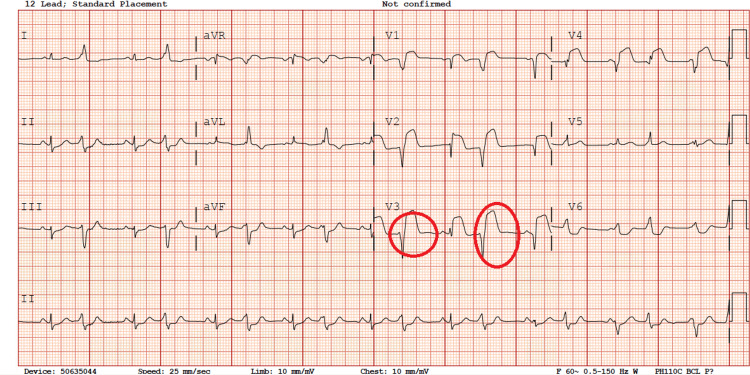
First ECG showing bigeminy (highlighted by red circles)

Upon presentation, vital signs revealed a fever of 100.7°F, heart rate of 94 beats per minute, respiratory rate of 22 breaths per minute, and oxygen saturation of 100% on room air. Initial blood pressure measurement was 93/55 mmHg. Physical examination indicated the patient appeared toxic and mildly distressed, with a slightly delayed capillary refill time of three seconds. The patient received a normal saline bolus and intravenous medications, including famotidine, ondansetron, and oral Maalox. Acetaminophen was administered for fever control. A comprehensive respiratory viral pathogen panel, including COVID-19, returned negative results. Complete blood count and chemistry panels were unremarkable. The initial electrocardiogram (ECG) conducted in the emergency department revealed evidence of bigeminy (Figure 1). Cardiac troponin levels were elevated at 27.78 ng/ml and 21.46 ng/ml, b-type natriuretic peptide (BNP) level was 656 pg/mL, D-dimer level was 428 ng/mL, ferritin level was 84 ng/mL, C-reactive protein (CRP) level was 4.19 mg/L, electrolyte levels were within normal range, and aspartate aminotransferase (AST) level was elevated to 168 U/L. COVID immunoglobulin G (IgG) was positive to 21 (AU/ml) (<=9.99 non-reactive). Consultations with pediatric cardiology and infectious disease specialists were initiated. Maintenance intravenous fluids were commenced, with ibuprofen administered every six hours with concurrent ECG monitoring every six hours.

**Figure 2:**
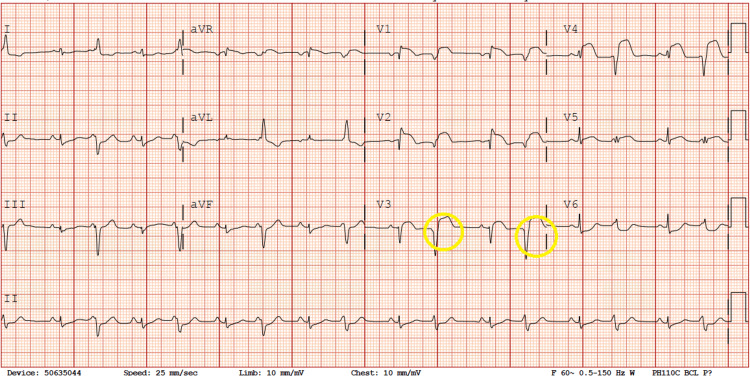
Second ECG showing AV blocks, bigeminy, and premature ventricular complexes (yellow circles)

Bedside echocardiography in the ED revealed a mildly depressed ejection fraction. Subsequently, the patient was admitted to the pediatric intensive care unit (PICU) for cardiac monitoring. Upon admission to the PICU, she was placed on continuous cardiac telemetry and started on a dopamine drip at 3 mcg/kg/min due to hypotension. A follow-up electrocardiogram (ECG) performed six hours later revealed evidence of conduction blocks with bigeminy (Figure 2). Shortly after, the patient experienced a self-terminated episode of narrow complex tachycardia lasting approximately eight minutes. Although the patient remained conscious and alert throughout the episode, she reported discomfort, the decision was made to initiate a transfer of the patient to a tertiary care facility with specialized cardiac intensive care and ECMO capabilities.

**Figure 3:**
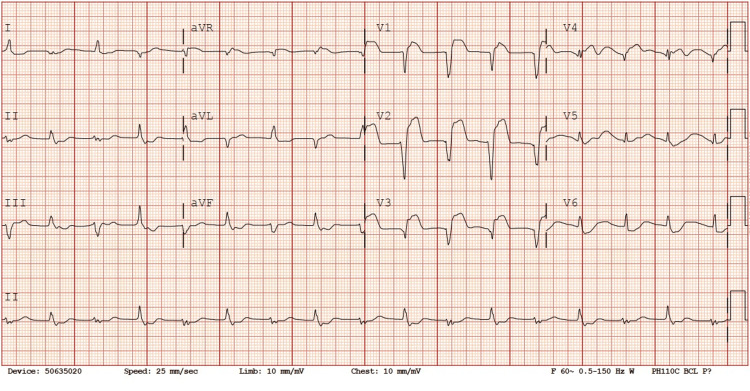
Third ECG showing indeterminate arrhythmias

She continued to have repeat episodes of erratic rhythms mostly presenting as supraventricular tachycardias (SVTs) up to HR of 230s while awaiting transfer. An electrophysiologist was consulted who recommended several rate control modalities that involved three doses of adenosine; the first two boluses were 1 mg/kg/dose and a third dose of 2 mg/kg, all doses were given but without effect. She was then given two loading doses of lidocaine at 1 mg/kg, which temporarily brought her HR down to the 150s, and then placed on a continuous lidocaine infusion started at 20 mcg/kg/min and eventually titrated up to 45 mcg/kg/min. A repeat ECHO in the ICU about seven hours after the first ECHO showed a moderate to severely depressed biventricular function (left greater than right) for which she was placed on a dobutamine drip at 2.5 mcg/kg/min. Arterial blood gases showed a stable pH of 7.4 with mild metabolic acidosis and respiratory compensation with pCO_2_ in the 20s and lactate of 1.5-1.8. stable. While awaiting transfer, the patient continued to have repeat episodes of narrow complex tachycardia despite the lidocaine infusion (Figure 3). She was placed on a bilevel positive airway pressure (BiPAP) for LV function to decrease preload and LV afterload, and she was transferred on both dopamine and dobutamine drips. Unfortunately, shortly after she arrived at the tertiary facility, the patient went into cardiac arrest, and despite multiple resuscitation attempts, unsuccessful outcomes were encountered.

## Discussion

MIS-C is a systemic inflammatory condition triggered by a COVID-19 infection, typically emerging following an acute infection. The Centers for Disease Control and Prevention (CDC) criteria for diagnosing MIS-C include all three of the following: A. Individuals under 21 years of age presenting with fever, laboratory evidence of inflammation, and signs of clinically severe illness necessitating hospitalization, with involvement of two or more organ systems (cardiac, renal, respiratory, hematologic, gastrointestinal, dermatologic, or neurological); B. Absence of alternative plausible diagnoses; C. Positive testing for current or recent SARS-CoV-2 (COVID-19) infection by reverse transcription-polymerase chain reaction (RT-PCR), serology, or antigen test; or exposure to COVID-19 within four weeks before symptom onset [1-4].

Cardiac involvement stands out as a prominent feature of MIS-C and is associated with significant morbidity and mortality if not promptly addressed. The cardiac manifestations of MIS-C can be categorized into laboratory, electrocardiography (ECG), echocardiography, and clinical changes. Common manifestations include a. Laboratory changes; elevated pro-BNP, BNP, and/or elevated troponin; b. ECG changes; nonspecific ECG changes, conduction abnormalities, and arrhythmias; c. Echocardiography changes: myocardial dysfunction, and coronary artery dilatation, as well as other echocardiographic findings such as pericardial effusions, mitral regurgitation, valvular insufficiency, and regional wall abnormalities [5,6].

While the majority of pediatric COVID-19 cases present with mild symptoms, a subset may develop MIS-C as a sequela. The etiology of MIS-C remains unclear, although genetic predisposition may play a role. Current epidemiological and clinical data have not definitively linked MIS-C development to demographic factors such as age, race, gender, or socioeconomic status. Further comprehensive studies are warranted to better understand the pathogenesis and risk factors associated with MIS-C [7].

In the literature, arrhythmias associated with MISC have been in large part benign but in rare cases like the case described above, they can lead to fatal consequences when uncontrollable arrhythmias manifest. Arrhythmic complications constitute a significant component of the cardiac manifestations observed in MIS-C. These rhythm disturbances present clinical challenges and can contribute to adverse patient outcomes. A thorough understanding of the spectrum of arrhythmic complications in MIS-C is essential for optimizing patient management and outcomes [5-7].

A diverse array of arrhythmias has been reported in MIS-C patients, ranging from transient benign disturbances to severe ventricular arrhythmias. Common manifestations include sinus tachycardia, supraventricular tachycardia, atrial fibrillation, atrial flutter, and various degrees of atrioventricular (AV) conduction abnormalities. While some arrhythmias may spontaneously resolve with treatment addressing the underlying inflammatory process, others may persist or worsen, necessitating interventions to restore normal cardiac rhythm and mitigate adverse outcomes [7,8].

In a case series published in 2020 by Whittaker et al., which examined the clinical characteristics of 58 hospitalized children meeting the diagnostic criteria for MIS-C, it was noted that arrhythmic complications occurred in four patients. Specifically, one patient exhibited first-degree atrioventricular block accompanied by frequent supraventricular ectopic beats, and another experienced intractable broad complex tachycardia associated with reduced cardiac output, necessitating the use of extracorporeal membrane oxygenation (ECMO). Additionally, one patient presented with atrial fibrillation managed with amiodarone while another exhibited second-degree heart block, which resolved spontaneously without intervention [9].

The precise pathogenetic mechanism underlying the cardiovascular effects of MIS-C remains incompletely elucidated. Nonetheless, several plausible hypotheses have been proposed. The pathophysiologic pathways leading to cardiac injury in COVID-19 are multifaceted and may involve direct viral myocardial injury, hypoxia, hypotension, elevated inflammatory status, down-regulation of angiotensin-converting enzyme 2 (ACE2) receptors, drug-induced toxicity, and endogenous catecholamine adrenergic activity, among others [10-12].

Many of these hypotheses regarding the pathogenesis of cardiac complications are informed by insights gained from previous experiences with SARS-CoV, MERS-CoV, and H1N1 influenza. Cardiac conduction abnormalities and arrhythmic complications are theorized to arise from direct myocardial damage affecting the cells of the conduction system and potential myocardial scarring leading to conduction defects and subsequent arrhythmic complications. Furthermore, the proinflammatory milieu associated with MIS-C can exacerbate pre-existing arrhythmogenic substrates or predispose previously healthy individuals to arrhythmic events [11,12].

In cases of simple arrhythmias, rate control antiarrhythmics may be suitable like beta-blockers. Standard antiarrhythmics can be used for short-term management but in cases where arrhythmias progress quick access to advanced cardiac care with ECMO is indicated as in viral myocarditis. Ultimately, the most important principle in management is allowing time for myocardial healing and recovery [13].

Standard and conventional guidelines should be followed to manage arrhythmias using various classes of antiarrhythmics as indicated. The framework for the management of COVID-19/MIS-C-associated arrhythmia is similar in management to viral myocarditis with symptomatic support being vital to management as a bridge to allow for myocardial recovery just as with any viral myocardial infarction [14].

After control of rhythm abnormalities and rate issues standard management of MISC is indicated. Current evidence and guidelines often mirror strategies employed in Kawasaki disease (KD), which is primarily focused on controlling and mitigating inflammation. These treatment strategies are typically guided by institutional protocols with intravenous immunoglobulin (IVIg) or steroids. Some institutions recommend a combination of both [15].

Data on the resolution of arrhythmia vary. Some case studies and cohort studies have shown a range of 1-5; for example, the study by Choi et al. described the resolution of AV blocks within one to five days with MIS-C treatments as described above. The American College of Rheumatology suggests that all children with MIS-C undergo repeat echocardiography within 7-14 days and then at 4-6 weeks following initial presentation. For patients demonstrating cardiac involvement during the acute phase, consideration should be given to an additional echocardiogram one year post-MIS-C diagnosis [15].

While antiviral therapy is not commonly employed for MIS-C, institutions may consider the use of remdesivir based on the severity of acute-phase symptoms. Sperotto et al. advocate for comprehensive follow-up of MIS-C patients with cardiac involvement, recommending evaluations at 7-10 days, 4-6 weeks, 4-6 months, and 9-12 months post-diagnosis. This includes monitoring of inflammatory markers in the immediate post-acute phase and serial echocardiograms during subsequent cardiology visits. Exercise restrictions are advised per the 2021 American Heart Association guidelines for three to six months after MISC-associated myocarditis to minimize the risk of life‐threatening arrhythmia, with Holter monitoring and exercise stress testing performed in athletes before they return to sports [16].

## Conclusions

Current treatment strategies have proven effective at resolving many of these cardiac findings, but there is still room for improvement. Close disease surveillance is ongoing and will further characterize the cardiac manifestations and potential sequelae of MIS-C. Multi-center collaborations and harmonized registries are key to understanding the natural history, refining diagnostic criteria, developing risk stratification algorithms, and determining best management.
